# Macrophages Interaction and MicroRNA Interplay in the Modulation of Cancer Development and Metastasis

**DOI:** 10.3389/fimmu.2020.00870

**Published:** 2020-05-12

**Authors:** Ioana Iurca, Alexandru Tirpe, Alina-Andreea Zimta, Cristian Moldovan, Diana Gulei, Ondřej Slabý, Gerolama Condorelli, Ioana Berindan-Neagoe

**Affiliations:** ^1^Tumor Biology Department, The Oncology Institute “Prof. Dr. Ion Chiricuta”, Cluj-Napoca, Romania; ^2^Research Center for Functional Genomics, Biomedicine and Translational Medicine, Iuliu Hatieganu University of Medicine and Pharmacy, Cluj-Napoca, Romania; ^3^Faculty of Medicine, Iuliu Hatieganu University of Medicine and Pharmacy, Cluj-Napoca, Romania; ^4^Research Center for Advanced Medicine—Medfuture, Iuliu Hatieganu University of Medicine and Pharmacy, Cluj-Napoca, Romania; ^5^Centre for Molecular Medicine, Central European Institute of Technology, Masaryk University, Brno, Czech Republic; ^6^Department of Comprehensive Cancer Care, Faculty of Medicine, Masaryk Memorial Cancer Institute, Masaryk University, Brno, Czech Republic; ^7^Department of Molecular Medicine and Medical Biotechnology, Federico II University of Naples, Naples, Italy; ^8^Department of Functional Genomics and Experimental Pathology, The Oncology Institute “Prof. Dr. Ion Chiricuta,” Cluj-Napoca, Romania

**Keywords:** macrophage, cancer, metastasis, microRNA, invasion

## Abstract

Advancement in cancer research has shown that the tumor microenvironment plays a crucial role in the installation, progression, and dissemination of cancer cells. Among the heterogeneous panel of cells within the malignant microenvironment are tumor-associated macrophages that are sustaining the malignant cells through strict feedback mechanisms and spatial distribution. Considering that the presence of metastasis is one of the main feature associated with decreased survival rates among patients, in the present article we briefly present the involvement of tumor-associated macrophages in the hallmarks of metastasis and their microRNA-related regulation with a focus on lung cancer in order to coordinate the vast information under one pathology. As shown, these cells have emerged as coordinators of immunosuppression, angiogenesis and lymphangiogenesis, vessel intravasation and extravasation of cancer cells, and premetastatic niche formation, transforming the macrophages in potential therapeutic targets and also prognostic markers according to their density within the tumor and polarization phenotype. An indirect therapeutic approach on tumor-associated macrophages can be also represented by regulation of microRNAs involved in their polarization and implicit oncogenic features. Examples of these microRNAs consist in the highly studied miR-21 and miR-155, but also other microRNA with less feedback in the literature: miR-1207-5p, miR-193b, miR-320a, and others.

## Introduction

In 2018, there were an estimated number of 18.1 million new cancer cases, with a staggering 9.6 million cancer-related deaths. According to GLOBOCAN 2018, lung cancer was the most commonly diagnosed cancer and the prime cause of cancer death, marking it as the leading malignancy in terms of incidence and mortality (1). Considering the aforementioned details, we will exemplify the role of macrophages with a focus on lung cancer in order to simplify and coordinate the vast information under a sole oncological pathology. However, important functions of macrophages are highlighted in other malignancies as well, with translational values along the oncology sector.

In the dramatic evolution of lung cancer, the tumor microenvironment (TME) plays a vital role in its development, from the initiation phase, progression, and culminating with metastasis (2). A key component of the TME in cancer is represented by tumor-associated macrophages (TAMs), elements generally associated with a poor prognosis in the neoplastic disease as objectified by Cassetta and Pollard (3). Oncological studies infer that TAMs play a role in all stages of the metastatic process (4, 5). In most malignancies, the main principle that leads to the genesis of TAMs is the recruitment of monocytes from the circulation and their differentiation under TME conditions (6). Different stimuli can lead to the polarization of macrophages into two main categories: classically activated M1 macrophages or alternatively activated M2 macrophages, each with various properties. The classical M1 macrophages are driven by the Th1 cytokine interferon (IFN)-γ and secrete a number of interleukins (ILs), including IL-6, IL-12, and IL-23, as well as tumor necrosis factor (TNF)-α. In contrast, the M2 phenotype macrophages increase the expression of mannose receptors (MR, CD206), CD163, and scavenger receptors and produce the immunosuppressive IL-10. The M2 phenotype is characterized by anti-inflammatory and pro-oncogenic activities (7–9). Each of these subtypes has properties that may influence cancer progression.

A retrospective study by Ma et al. (10) which analyzed 50 patients with non-small cell lung cancer (NSCLC) found that ~70% of TAMs were M2-polarized macrophages and the remaining 30% were M1-polarized, highlighting the large proportion of the alternatively activated M2 macrophages. The team established that the M2 macrophage densities were not associated with patient's survival time. Contrarily, the M1 macrophage densities were associated in a positive manner with patient survival time in a univariate analysis (*p* < 0.01 or 0.001). In the same study, the team showed that M1 macrophage density in the NSCLC tumor islets was an independent predictor of the patient's survival time (10).

## Macrophages Interplay During Cancer Progression: From Primary Tumor to Metastatic Niche Formation

It is now known that TAMs are involved in a wide variety of mechanisms related to tumorigenesis and are essential components of the malignant microenvironment (11). Once stimulated, TAMs can influence the surrounding cells through secretion of growth factors, proteolytic enzymes, cytokines, and inflammatory substrates that further contribute to different tumor promoting mechanisms: immunosuppression (PD-L1, PD-L2, CD80, CD86, IL-10, TGF-β, Arginase-1, prostaglandins), cancer stem cells (TGF-β1, IL-10, MFG-E8, IL-6), epithelial to mesenchymal transition (EMT) (TLR4/IL-10 signaling, TGF-β) (11). According to Condeelis and Pollard, other processes that lead to cancer progression are angiogenesis (VEGF, ADM, PDGF, MMPs, TGF-β, CCLa, SEMA3A), intravasation, and extravasation (EGF, CCL-18, P2Y2 receptor), migration and invasion (MMPs, serine proteases, cathepsins, MIP-1β, EGF) (11, 12).

The localization and the density of TAMs within the tumor microenvironment have functional meaning upon cancer evolution and also progressive clinical and pathological features. Through secretion of chemoattractants within the local malignant environments, TAMs are recruited at the invasive edge of the tumors or the perivascular areas; hereby these cells can positively modulate the invasion process (13). Specifically, in the early phases of cancer evolution, the tumor cells take advantage of the TAMs' capacity of matrix remodeling; in this sense, TAMs are mainly found at the spots of basement-membrane breakdown, allowing the tumor cells to invade the surrounding stroma (14). Through multiple studies involving the density of macrophages at the tumor spots, it was generally observed that tumors infiltrated with macrophages have increased metastatic potential (15). Specifically for breast cancer, colony stimulating factor 1 (CSF-1) is expressed in the majority of human breast malignancies and is involved in the recruitment of macrophages that are further implicated in the invasion of the breast cancer cells within the stroma and to a subsequent access to vasculature. Patients with high CSF-1 expression are associated with low survival (16). Experimental stimulation of cells with epidermal growth factor (EGF) or CSF-1 determined simultaneous migration of both tumor cells and macrophages even in the case that only macrophages express the receptor for CSF-1 and the receptor of EGF is present strictly in tumor cells. No matter the combination between the two molecules, synergistic migration was observed, confirming a functional interaction between the two types of cells during cell invasion and the presence of a paracrine signaling within the EGF/CSF-1 axis (15); the proposed mechanism consists in initial migration of cells toward one another and concomitant penetration of a dense collagen matrix. The interaction between macrophages, tumor cells, and blood vessels was also confirmed through multiphoton imaging (17). The carcinoma cells present a polarized movement along the collagen fibers; these fibers are sustaining through convergence the blood vessel at the center, where macrophage along tumor cells are directed (17). The further intravasation of the tumor cells within the blood vessel is also mediated by macrophages; inhibition of the paracrine loop between CSF-1 and EGF through silencing of epidermal growth factor receptor (EGFR) signaling results in blocked intravasation (15). The correlation between the density of macrophages and the spots of intense vasculature is also assuming a role of these cells in tumor angiogenesis. Macrophages are also densely organized in hypoxic regions of the tumor (18) where the same hypoxic environment is stimulating the cells in overexpressing hypoxia inducible factor (HIF) transcription factors that further act upon angiogenic factors, including VEGF, with the direct effect of stimulating blood vessel development (19); other molecules secreted by macrophages include the platelet-derived growth factor (PDGF), adrenomedullin (ADM), matrix metalloproteinases (MMPs), and TGF-β (11). Specifically, in cervical cancer, inhibition of MMP-9 in macrophages within mice models impaired the secretion of VEGF and subsequently the formation of blood vessels and tumor growth (20). In Merkel cell carcinoma, a neuroendocrine skin malignancy, TAMs can stimulate lymphovascularization through secretion of VEGF-C (21). In hypoxic conditions, in dependency to HIF-1α secretion, TAMs can express PD-L1 (22) that functions as a ligand for the inhibitory receptor programmed cell death protein 1 (PD-1) and inhibits the activity of T-cells; therefore, macrophages are also contributing to the process of immune escape, protecting the malignant cells from the cytotoxic activity of T-cells (23). Other molecules secreted by TAMs include IL-10 and TGF-β that impair the activity of T-cells and also the maturation of dendritic cells (DCs) (24–26).

**Figure 1:**
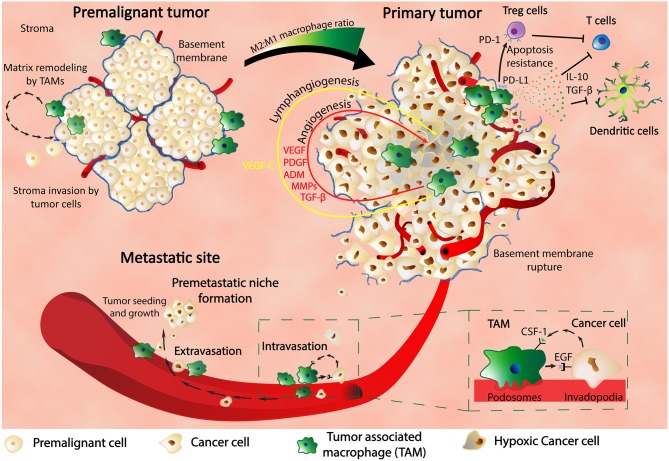
Macrophages are key players in cancer. In the premalignant tumor, TAMs find themselves at spots of basement membrane breakdown, infiltrating the stroma and remodeling the surrounding matrix. In the primary tumor, macrophages are found in hypoxic regions where HIF-1α stimulates TAMs to produce various pro-malignant factors such as VEGF, PDGF, ADM, MMPs, and TGF-β, thus inducing angiogenesis, lymphangiogenesis, metastasis, and cancer progression. TAMs also express PD-L1 that binds to PD-1 of Treg cells and determine their apoptosis resistance with further inhibitory effect upon T cells. Production of IL-10 and TGF-β by TAMs are another way of inhibiting the activity of the immune systems: T cell activity and dendritic cells development. TAMs are in close contact with the cancer cells through the blood vessel intravasation and extravasation and also influence each other through feedback mechanisms composed of CSF-1 and EGF and their receptors. Finally, TAMs are contributing to the formation of the premetastatic niche and also to the growth and proliferation of the metastatic tumors.

As shown, TAMs are critical players for tumor survival and development at the primary site and also intravasation into blood vessels; furthermore, the same cells are also involved in the extravasation process and pre-metastatic niche (PMN) formation (Figure 1). Previous studies in a metastatic breast cancer animal model showed through imaging techniques that macrophages are recruited to the extravasation sites (pulmonary metastatic sites) and form a close contact with the cells about to populate metastatic spots. The same macrophages are essentials for the metastatic seeding and growth as shown by the macrophages depletion experiments that inhibited further metastatic growth even after earlier formation of metastatic colonies (27).

The coordination of macrophages with cancer development is also demonstrated through their specific transcriptome: TAMs poses different signatures between cancer types (breast and endometrial malignancies) and also compared with the progenitor monocytes and corresponding resident macrophages. Therefore, this functional interaction is actively coordinated according to specific malignant parameters, including location and subtype. Cassetta et al. (28) identified a 37-gene TAM signature that is correlated with aggressive subtypes of breast malignancies and associated with shorter survival time.

## Induction and Interaction of Macrophage Polarization through MicroRNAs

MicroRNAs (miRNAs) are a type of small non-coding RNAs (ncRNAs) with an approximate length of 19–25 nucleotides that modulate gene expression, along with a diverse number of pathways (19, 29–32). These modulatory molecules are involved in a large variety of biological processes such as cell cycle, differentiation, and in immunity. Understandably, miRNAs are an integrative part of the pathogenic chain in a number of pathologies, including cancer, where these entities can act either as tumor suppressors miRNAs or oncomiRNAs (33–35).

MiRNAs are known regulators of macrophage polarization. The mechanism behind these modulatory effects include the targeting of a number of transcription factors and molecules that drive the main signaling cascades in macrophage polarization, such as the IRF/STAT pathways (36). MiRNAs have a heterogeneous profile; while some induce the classical macrophage activation, M1, others drive the alternative activation toward the M2 phenotype. Next, a summary of miRNAs being of importance in modulating the polarization state of macrophages in cancer, with a focus on lung cancer is presented.

MicroRNA-21 (miR-21) is a known promoter of the neoplastic/ tumor process (37, 38). Canfran-Duque et al. (39) showed that miR-21 downregulation favors M1 polarization, thus increasing the anti-tumoral effect. In a 2019 study by Ren et al., increased miR-21-5p delivery by extracellular vesicles secreted by mesenchymal stem cells (MSC-EV) after hypoxia pre-challenge was shown to reduce PTEN expression, leading to Akt and STAT3 activation and thus stimulating M2 polarization (40). Several other studies suggest a key role of miR-21 in the transition from a M1 to M2 phenotype (41, 42). MiR-21 was suggested as a prognostic factor in lung cancer, as it was associated with poor clinical outcome, with a negative impact on overall survival (OS) in NSCLC [HR = 2.32; 95% CI (1.17–4.62), *p* < 0.05], as well as recurrence-free survival (RFS)/cancer-specific survival (CSS) in lung AC [HR = 2.43; 95% CI (1.67–3.54), *p* < 0.001] (37).

A study by Hsu et al. on human AC cell lines NCI-H1437, NCI-H1792, NCI-H2087, human embryonic kidney HEK293 plus CL1-5 cells (6) showed that extracellular vesicles (EVs) containing **miR-103a**, a hypoxia-responsive miRNA (43), can be vectored from hypoxic lung cancer cells to macrophages, with an inductive effect toward immunosuppressive M2 polarization. EV miR-103a-derived macrophages show high levels of VEGF and angiopoietin-1 which stimulate neoangiogenesis, cancer migration and invasion. Furthermore, high EV miR-103a levels are detected in the sera of lung cancer patients (6). Mechanistically, miR-103a downregulates PTEN, a well-known tumor suppressor that is inactivated in a number of malignancies, including lung cancer (44). PTEN is involved in immunity modulation by its interaction with the PI3k/Akt pathway; inactivation or deficient PTEN promotes M2-type polarization (45–47); PTEN inhibition increases Akt and STAT3, which triggers a build-up of CD163^+^CD206^high^HLA-DR^low^ cells, as well as expression of pro-oncogenic factors. Hsu et al. (6) concluded that tumor hypoxia triggers a switch on tumor-suppressing macrophages toward a tumor-promoting state by secreting miR-103a EVs; these EVs target PTEN and activate PI3k/Akt and STAT3 pathway.

Another combined *in vitro* and *in vivo* 2016 study by Dang et al. identified CSF-1 as a miR-1207-5p target on A549 cells. CSF-1 is able to stimulate cancer cells directly or indirectly by modulating the host immune system. TAM proliferation is stimulated by CSF-1. These macrophages have wide effects, including tumor growth, angiogenesis and ECM lysis, as mentioned beforehand. The same team evaluated the effects of miR-1207-5p on macrophage function in d-THP1 cells. They revealed that miR-1207-5p increased M1 phenotype cytokines (IL-12, IL-23) and decreased M2 phenotype characteristics (IL-10, VEGF). Dang et al. concluded that miR-1207-5p can inhibit A549 lung cancer cell proliferation, migration and invasion, as well as HUVEC tube formation. MiR-1207-5p is also able to downregulate STAT3 and Akt signaling, as well as IL-10, CCL5, CXCL10 which are downstream targets of STAT3-Akt. MiR-1207-5p also regulates a number of EMT-involved molecules such as SNAIL, SMAD2, SMAD3, SMAD7, Vimentin, and ZEB1 with an inhibitory role in tumor invasion and metastasis. The same team used nude mouse xenograft model to support that miR-1207-5p suppresses lung cancer cell metastasis *in vivo*. Lastly, Dang et al. found miR-1207-5p expression to be downregulated in NSCLC specimens, with an upregulated CSF-1 expression in NSCLC tissues compared to non-cancerous lung tissue (48).

A study by Huang et al. (49) showed that cypermethrin (CYM), a type II pyrethroid, promoted a shift toward M2 macrophage polarization by downregulating miR-155. MiR-155 (50) downregulation enhanced Bcl-6 expression with a consecutive reduction of the mitogen-activated protein kinase 4 (MKK4) and an inhibition of cJun NH_2_-terminal kinase (JNK), a central member of the MAPK families. Huang's team found that overexpression of Bcl-6 upregulated M2-associated gene expressions, including Arg1 and Mgl2, whilst downregulating M1-associated genes—TNF-α, IL-6, iNOS. Thus, downregulation of miR-155 is able to promote macrophage polarization shift from M1 to M2. The same authors proved that CYM-treated macrophages induced Lewis lung cancer cells metastasis both *in vitro* and *in vivo* (49). Concomitantly, a screening on miRs expression profiles by Graff et al. (51) found miR-155 in both M1 and M2b-polarized macrophages.

However, there are several other miRNAs that are involved in macrophage polarization. Supplementary Table 1 presents specific miRNAs, together with their pathological expression, target genes, and effect upon macrophage phenotype and implicit cancer development. Among this miRNAs, miR-130, miR-320a, miR-125a, miR-26a, miR-27a, miR-23a, miR-132, miR-222-3p, miR-193b, and miR-29b (and also the ones presented above), have been associated in the literature with macrophage dynamics in the context of solid malignancies. Specifically, we describe how the expression of these miRNAs influence their targets and the overall effect on macrophage polarization as observed in the cited studies, and possible therapeutic context focused on the modulation of the non-coding RNAs toward limitation of metastasis and cancer progression.

## Means of Communication Between M1/M2 Macrophages and Cancer Cells

Cancer cells and local macrophages have an intricate way of communication that allows M2 polarization, local immunosuppression, cancer cell survival, and spreading. There is the cell-to-cell contact through receptor-ligand interaction, secretion in the local environment of factors as free molecules or encapsulated in the exosomes which are nano-sized vesicles involved in intercellular communication (52), and distant spreading of anti-inflammatory/tumor promoting factors.

Exosomes generated from cancer cells, being charged with oncogenic molecules, induce the secretion of immune mediators such as IL-6, IL-10, CXCR4, and C-C Motif Chemokine Ligand 2 (CCL2), and subsequently lead to TAM-like phenotype transition, while their stimulation is insufficient to generate macrophage polarization to either M1 or M2 phenotype (53). The exosomes secreted by cancer cells versus the ones secreted by physiological cells deliver a specific message to macrophages. The breast cancer exosomes induce the activation of NF-κB and the overexpression of IL-6, TNFα, GCSF, and CCL2 in macrophages located in the brain and lung through the binding of Toll-like receptor 2 (TLR2) or MyD88 (54).

Sometimes the effects of the communication between macrophages and tumor cells are not specific. M0 (macrophages in resting state), M1, and M2 macrophages can enhance the intracellular level of ubiquitin-specific protease (USP17) in Lewis lung cancer cells, which further causes the upregulation of USP17 to both pro-inflammatory (TNF-α, IL-1β, IL-6, IL-8, CXCL12) and the anti-inflammatory factors IL-4 and IL-10. USP overexpression maintains cancer cell stemness resulting in an enhanced adaptability of cancer cells (55).

Lung cancer cells and macrophages seem to be able to establish an interaction similar to that of PD-1–PD-L1, meaning that malignant cells in small cell lung cancer express the CD47 antigen when they bind to the SIRPα receptor, leading to the macrophage loss of capability to phagocyte the cancer cells. The macrophages exhibit a phenotype that is specific for neither M1, nor M2. These are termed “M2-like” macrophages, because while they are partially anti-inflammatory, they are not a specific M2. The anti-CD47 treatment significantly reduced *in vivo* tumor growth (56). Moreover, a study of malignant pleural effusion demonstrated that macrophages express PD-L1 at low intensity in comparison with cancer cells and that this low level is associated with a poor prognosis. Moreover, PD-L1 overexpression in macrophages is associated with polarization toward the M1 phenotype and an increase in survival rate (57).

The M2 macrophages secrete IL-10 in the extracellular media which binds to the IL-10RA, causing the activation of JAK1, STAT1, STAT3, STAT6, NF-κB, and Notch in lung cancer cells. This further results in the elevated transcription level of the cancer stem cell markers (SOX2, Oct4, c-Myc). The NSCLC cells exposed to IL-10 undergo EMT by overexpressing Vimentin (VIM) and N-Cadherin (N-CAD). In lung cancer, tumor growth and patient survival is closely linked to the IL-10/JAK1 axis activation (58).

Through extracellular vesicle or soluble factors, the M2 macrophages can increase the expression of VEGF, MMP-2, and MMP-9 in NSCLC cells and promote migration, while the M1 macrophages have the exact opposite effect (59). NSCLC that was exposed to increasing concentrations of cisplatin or doxorubicin until reaching chemoresistance showed overexpression of NF-κB that leads to the secretion in the extracellular environment of IL-34. This cytokine binds to colony stimulating factor 1 receptor (CSF-1R) in the monocytes, directing their transformation toward the M2 phenotype and stimulating the secretion of anti-inflammatory, tumor-promoting factors IL-10 and TGF-β. IL-34 is also internalized in lung cancer cells where it stimulates the Akt pathway activation, thus forming an autocrine mean of chemoresistance (60).

The lung cancer cells can, in their turn, induce the switch of monocyte-derived macrophages to an M2 phenotype through soluble factors or extracellular vesicles. This switch is marked by increased secretion of IL-10 and decreased level of IL-12 and TNF-α by these malignant cells. If the M2 transformation is suppressed, then the cancer cells show decreased proliferation and tumor growth through suppression of STAT3 (61). The lung cancer cells can determine the local recruitment of macrophages through excretion of VEGF-C in the extracellular media. This factor is recognized by VEGFR3 in macrophages, where it activates the SRC/p38 pathway. These macrophages do not show M1 or M2 specificities, instead they stimulate the *in vivo* metastasis of tumor cells (62).

The lung is a frequent site of metastasis generated from various primary tumors. These tumors secrete extracellular vesicles that are engulfed by the local lung macrophages, overexpressing CCL2, and recruiting monocytes. The monocytes are later transformed into a type of macrophages that express Arginine 1 on their surface and secrete CD206 and IL-10, thus rendering them a M2 phenotype. These macrophages sustain local fibrosis, creating an anti-inflammatory milieu proper for engraftment of a new tumor (63).

Cancer-derived exosomes also have the opposite effect regarding metastatic niche formation in the lung, through the generation of “non-metastatic exosomes” by the cancer cells from primary melanoma tumors with less invasive capacity. These exosomes activate the migration of Ly6Clow patrolling monocytes (PMO) from bone marrow to lungs and phagocytosis of migrating cancer cells in a TNF-related apoptosis-inducing ligand (TRAIL)-dependent manner (64).

The macrophage-derived exosomes are also excellent means of drug delivery. Through biotechnological engineering of the exosomes they become hollow, but highly specific nanoparticles. The exosomes isolated from the bone marrow of C57BL/6 mice express Anisamide, a ligand of Sigma receptor which is present on the surface of lung cancer cells. These exosomes were loaded with paclitaxel, a chemotherapeutic agent. These options of chemotherapy delivery resulted in a targeted decreased viability of cancer cells (65). The pro-inflammatory ability of M1 macrophages exosomes can efficiently be used in anti-cancer therapy. Upon *in vivo* delivery, the M1-derived exosomes are taken up by the local lymph nodes and combined with local macrophage and dendritic cells. The M1 exosomes contain pro-inflammatory cytokines IL-6, IL-12, and IFN-γ (66). In Caveolin-2 knock-out mice the M1 macrophages were the predominant phenotype, causing the lung tumors to have a smaller size in comparison with wild-type mice. This membrane protein might be a method of macrophage-malignant cell interaction (67).

Lung cancer cells found in close contact with M2 macrophages present increased proliferation and migration capacity. They secrete CCL2 and C-X3-C Motif Chemokine Ligand (CX3CL1) that attract circulating monocytes and together with overexpression of the CCR2 and CX3CR1 receptors, also cause the M2 polarization of macrophages (68). Moreover, as stated above, cancer cells secrete IL-34 that activates the colony-stimulating factor receptor (CSFR) found on the macrophage surface thus facilitating their polarization to M2 phenotype, specifically for TAMs. (60). In their turn, M2 macrophages secrete both pro-(IL-6) (69, 70) and anti-inflammatory cytokines (IL-10) (58) with oncogenic potential that sustain the EMT of lung cancer cells, through downregulation of E-CAD and upregulation of VIM, N-CAD. The role of M2 macrophage is supporting lung tumor progression not only by the modulation of malignant cell behavior, but also through the modulation of local immunity. For instance, the M2 macrophages decrease the local number of CD4^+^ and CD8^+^ T cells, without affecting their activation capacity (71). Table 1 summarizes the bidirectional changes between lung cancer cells and macrophages. These changes are an essential part of the modulative effects that TAMs have on corresponding cancer cells and vice versa. We describe the complex means by which these cells interact with each other (including receptors, exosomes, direct contact), along with the molecular and biological effects in order to integrate the information into a more comprehensive overall view.

**Table 1:** Means of communication, transmitted factors, and the exerted effects between macrophages and different lung malignant cell types.

**Type of cell**	**Cancer**	**Direction of communication**	**Means of communication**	**Transmitted factors**	**Effects—molecular**	**Effects—biological**	**References**
M2	LUAD (H1395 and H197)	Macrophage—lung cancer cells	Conditioned media was added (exosomes or free secreted factors)	N/A	CXCL17	Stimulation of spine metastasis	(72)
M2	NSCLC	Lung cancer cells—Macrophage	N/A	N/A	p38 (p-p38) which further increases HIF-1α	Hypoxia induces M2 polarization through p38	(73)
M2	Lewis lung carcinoma	Macrophage—lung cancer cells	Chemokine receptors CCR2 and CX3CR1 chemokine receptor	IL-1, MIP1α0, IL-6, CCL1, G-CSF upregulated in the system	CCR2 and CX3CR1 upregulation after IL-10 or MIP1α exposure, upregulation of MMPs, GF, VEGF	Aggressiveness of lung cancer cell increased more in direct contact with macrophage than in non-contact culture	(68)
M2	SCLC	Macrophage—lung cancer cells	Non-contact cell culture, culture media, soluble factors	IL-6	STAT3 activation	Lung cancer cell proliferation and invasion	(69)
TAMs	NSCLC	Macrophage—lung cancer cells	Contact *in vivo* tumor	TNF-α	Depletion of TAMs resulted in decreased GLUT1, PDK1, PDH, PGK, HK2, G6P, VEGFA, CA-9, NOS2; PD-L1	Increased glycolysis in cancer cells, decreased infiltrated T cells	(71)
M1/M2	Lewis lung carcinoma	Macrophage (M2)—lung cancer cells	Non-contact cell culture, culture media, soluble factors	pAMK	Upregulation of AMPKα in the M2 macrophage	Migration/invasion	(74)
SIRPα expressing macrophage	SCLC	Lung cancer cells—Macrophage	Direct contact through antigen (CD47—tumor) and receptor (SIRPα–macrophage)	N/A		M2-like phenotype, without capacity of phagocytosis	(56)
M2	NSCLC	Macrophage—lung cancer cells	Secreted cytokines in the culture media (IL-10)	IL-10	SOX2, Oct4, c-Myc, Vimentin, N-CAD - upregulation, phosphorylation of JAK1, STAT1/STAT3/STAT6, NOTCH1	Cancer stemness and EMT	(58)
M2	NSCLC	Macrophage—lung cancer cells	Secreted cytokines in the culture media IL-6	IL-6	E-CAD downregulation, VIM upregulation, β-catenin translocation in the nucleus, COX2, PGE2 upregulation	EMT	(70)
M2	NSCLC	Lung cancer cells—Macrophage	Supernatant from cancer cells	IL-34	Binding to the CSF-1R receptor, IL-10, and TGF-β overexpression	Polarization to anti-inflammatory phenotype, forms a feedback loop with cancer cells to sustain chemoresistance	(60)
M1	Murine lung carcinoma cell lines (LLC26 and CMT 167)	Lung cancer cells—Macrophage	N/A	N/A	N/A	The lack of Caveolin-2 enhances local polarization of M1 macrophage, which further stimulates the local acquisition of CD8^+^ and CD4^+^ T cells, leading to smaller tumors	(67)

## Conclusion

Approximately 70% of macrophages residing in lung tumors are anti-inflammatory M2 macrophages. The M2 macrophages are oncogenic, while the M1 macrophages are tumor suppressors. M2 macrophages are usually specialized in the suppression of local tumor immunity and acquisition of oncogenic features, commonly referred to as TAMs. The intratumoral distribution of M2 macrophages is centered on the basement membrane and the hypoxic region of the tumor core. On one side, the M2 macrophages sustain secretion of metalloproteinases promoting local invasion through the degradation of the basement membrane, and on the other side they cause an overproduction of pro-angiogenic factors such as VEGF as a response to hypoxic conditions. The miRNAs, through their multi-targeted activity are master regulators of macrophage polarization. In lung cancer, miR-21 and miR-103a stimulate M2 polarization and cause activation of the oncogenic pathway AKT-STAT3, miR-1207-5p, and miR-155 are tumor suppressor miRNAs that stimulate M1 polarization. The local macrophages and lung cancer cells communicate in different manners. Firstly, by cell to cell contact through CD47 (cancer cell) and SIRPα (macrophage) binding, CCR2, or CX3CR1 chemokine receptors; Secondly, through soluble factors or exosomes loaded in IL-10. The cancer cell-derived exosomes are also able to mediate the immunosuppressive effect of macrophages found at distant sites of the body through a process called metastatic niche preparation. To conclude, the future design of novel lung cancer therapies should consider polarization of macrophage to M1 phenotype through exogenous upregulation of miR-155 or miR-1207-5p, suppression of miR-21, or miR-103a, inhibition of IL-10 and agonist of CD47. The stimulation of M1 macrophage will have the advantage of creating a perpetuating downstream effect through activation of cytotoxic killing of cancer cells through the activity of CD4^+^, CD8^+^ T cells, followed by phagocytosis by macrophage and multi-level suppression of tumor advancement.

## Author Contributions

AT, DG, A-AZ, OS, GC, and IB-N contributed to conceptualization. II, AT, DG, and A-AZ contributed to writing and original draft preparation. All authors contributed to final editing. DG contributed to image conceptualization. CM contributed to image design. IB-N contributed to project administration and supervision.

## Conflict of Interest

The authors declare that the research was conducted in the absence of any commercial or financial relationships that could be construed as a potential conflict of interest.
