# Supplementary material for: Macrophages Interaction and MicroRNA Interplay in the Modulation of Cancer Development and Metastasis
Source: Front Immunol. 2020 May 12;11:870. doi: 10.3389/fimmu.2020.00870 (PMC7235377; doi:10.3389/fimmu.2020.00870)
Supplement: Supplementary file 1 [file Table_1.docx]

**Supplementary Table 1**. MiRNAs targeting a diverse palette of targets that modulate the inducement of macrophage polarization towards M1 or M2 phenotype in cancer

| **MicroRNA** | **MiRNA expression** | **Target** | **Effect** | **Ref** |
| --- | --- | --- | --- | --- |
| miR-130a | Downregulated | PPARγ | A study by Lin et al. reported PPARγ to be a target of miR-130a. Lin’s team demonstrated that miR-130a levels were high in M1-polarized macrophages compared to M2 macrophages. Transfection of macrophages with miR-130a mimic resulted in the downregulation of markers connected to the M2 phenotype, whilst an upregulation in M1 phenotype markers was observed. The same authors concluded that miR-130a has a critical role in the induction of pathological transition from an M2 phenotype towards an M1 phenotype. | (1) |
| miR-320a | Upregulated/Downregulated | STAT3  NRP-1  STAT4  IGF-1R  VDAC1 | MiR-320a is altered in several cancer types including lung cancer. In context, it is downregulated in NSCLC and acts as a tumor suppressor by directly regulating IGF-1R and VDAC1 expression. Concomitantly, miR-320a is involved in metastasis formation through downregulation of STAT3 and NRP-1. Overexpression of miR-320a in heavy-smoker derived macrophages induced an M2 macrophage phenotype which was confirmed by upregulation of macrophage scavenger receptor CD163 and CD206, indicating an alternative and pro-tumorigenic activation of these cells. Fortunato et al. proposed that miR-320a acts as a switch to M2 by downregulation of STAT4, a crucial factor involved in macrophage polarization. | (2-7) |
| miR-125a | Downregulated | FIH1  IRF4 | Zhao et al. found that miR-125a expression was induced following LPS + IFN-γ stimulation in bone-marrow derived macrophages. MiR-125a upregulated the M1-related markers IL-12, iNOS and TNF-α and promoted M1 polarization through FIH1 targeting. Concomitantly, miR-125a downregulated M2 marker MR and inhibited M2 polarization in an IRF4-dependent mechanism. | (8) |
| miR-26a | Downregulated | M-CSF | In a study by Chai et al., the authors examined mRNA expression of IL-12b and IL-23 in THP-1 cells stimulated by phorbol myristate acetate and treated with conditioned medium (CM) of HCC (hepatocellular carcinoma) cells in order to identify the action of miR-26a on macrophage polarization. The team found that THP-1 cells treated with CM of HepG2-miR-26a cells presented a higher level of M1 markers, suggesting an M1 phenotype, thus inferring that miR-26a has an effect on macrophage polarization towards M1. | (9) |
| miR-27a | Upregulated | IRF4  PPARγ  IL-10 | MiR-27a, member of the miR-23a/27a/24-2 cluster, is a responsive regulator for the M1 and M2b-polarized macrophages. Rahl et al. reported that miR-27a is able to regulate macrophage inflammatory response by targeting IL-10, indicating the important role that miR-27a may have in macrophage polarization. Ma et al. found that miR-27a inhibits M2 polarization by targeting and IRF4/PPARγ through a negative feedback loop. The same authors found that overexpression of the miR-23a/27a/24-2 cluster is an essential regulator of the macrophage response to M1 phenotype stimulation. | (10, 11) |
| miR-23a | Downregulated | A20  JAK1/STAT6 | A study by Ma et al. on 293T cells identified miR-23a as a promoter of M1 phenotype by targeting A20 through a negative feedback loop. Their experiment showed that miR-23a simultaneously activated the NF-κB pathway and lead to increased levels of M1-specific cytokines.  The same team determined that miR-23a, an integrating part of the miR-23a/27a/24-2 cluster, targeted JAK1/STAT6. This cluster is increased by M2 phenotype-derived cytokines; miR-23a represses M2-related transcription factors, thus inhibiting M2 polarization through a negative feedback loop. | (10) |
| miR-132 | Upregulated | Screening for miRs expression profiles  Acetylcholinesterase (AChE) | Identified in M2b-polarized macrophages.  MiR-132 is upregulated in azoxymethane/dextran sodium sulfate-induced colitis-associated colon cancer (CAC). Activation of aryl hydrocarbon receptor increased miR-132 expression and suppressed macrophage infiltration and pro-inflammatory cytokines, thus improving CAC severity. MiR-132 had an anti-inflammatory effect by targeting AChE. | (12, 13) |
| miR-222 | Upregulated/Downregulated | Screening for miRs expression profiles  SOCS3  CXCL12 | Identified in M2a and M2b-polarized macrophages.  In a study by Ying et al. using patient blood samples and the epithelial ovarian cancer (EOC) cell lines Skov3, A2780 and monocytic cell line U937, the EOC-derived exosomes activated TAMs and induced cancer progression. The team found that these exosomes were enriched in miR-222-3p and confirmed that overexpression of miR-222-3p induced macrophage polarization to an M2 phenotype by targeting SOCS3, thus modulating the SOCS3/STAT3 pathway.  In a breast cancer study, Li et al. found that miR-222 was downregulated in breast cancer sample TAMs. The same authors identified CXCL12 as a target of miR-222 and that overexpression of miR-222 inhibited chemotaxis of macrophages both *in vitro* and *in vivo*. | (12, 14, 15) |
| miR-193b | Downregulated | Screening for miRs expression profiles | Identified in M2a-polarized macrophages | (12) |
| miR-29b | Downregulated | Screening for miRs expression profiles | Experimental increase of miR-29b levels resulted in increased levels of the M1 phenotype marker CXCL9, IL-6 and TNF-α (M1/M2b phenotype markers). | (12) |
| miR-21-5p | Upregulated | PTEN | Delivery of miR-21-5p in MSC-EV after hypoxia pre-challenge showed that miR-21-5p targeted PTEN and lead to an Akt and STAT3 activation, thus stimulating M2 polarization. | (16) |
| miR-103a | Upregulated | PTEN | In a study on human AC cell lines NCI-H1437, NCI-H1792, NCI-H2087, human embryonic kidney HEK293 plus CL1-5 cells, miR-103a downregulated PTEN, followed by Akt and STAT3 activation and a subsequent switch to a M2 phenotype. | (17) |
| miR-1207-5p | Downregulated | CSF-1 | Dang et al. found that miR-1207-5p downregulates STAT3 and Akt signaling. The team’s evaluation of miR-1207-5p effects on macrophage function in d-THP1 cells revealed that miR-1207-5p increased M1 phenotype cytokines (IL-12, IL-23) and decreased M2 phenotype characteristics (IL-10, VEGF). | (18) |
| miR-155 | Downregulated | Bcl-6 | Downregulation of miR-155 promotes macrophage polarization shift from a M1 phenotype to a M2 phenotype by increasing Bcl-6 levels. This increase in Bcl-6 reduces MKK4 and inhibits JNK. | (19, 20) |

References

1. Lin L, Lin H, Wang L, Wang B, Hao X, Shi Y. miR-130a regulates macrophage polarization and is associated with non-small cell lung cancer. Oncology reports. 2015;34(6):3088-96.

2. Zhu H, Jiang X, Zhou X, Dong X, Xie K, Yang C, et al. Neuropilin-1 regulated by miR-320 contributes to the growth and metastasis of cholangiocarcinoma cells. Liver international : official journal of the International Association for the Study of the Liver. 2018;38(1):125-35.

3. Zhang G, Jiang G, Wang C, Zhong K, Zhang J, Xue Q, et al. Decreased expression of microRNA-320a promotes proliferation and invasion of non-small cell lung cancer cells by increasing VDAC1 expression. Oncotarget. 2016;7(31):49470-80.

4. Wang J, Shi C, Wang J, Cao L, Zhong L, Wang D. MicroRNA-320a is downregulated in non-small cell lung cancer and suppresses tumor cell growth and invasion by directly targeting insulin-like growth factor 1 receptor. Oncology letters. 2017;13(5):3247-52.

5. Lv Q, Hu JX, Li YJ, Xie N, Song DD, Zhao W, et al. MiR-320a effectively suppresses lung adenocarcinoma cell proliferation and metastasis by regulating STAT3 signals. Cancer biology & therapy. 2017;18(3):142-51.

6. Gordon S. Alternative activation of macrophages. Nature reviews Immunology. 2003;3(1):23-35.

7. Fortunato O, Borzi C, Milione M, Centonze G, Conte D, Boeri M, et al. Circulating mir-320a promotes immunosuppressive macrophages M2 phenotype associated with lung cancer risk. International journal of cancer. 2019;144(11):2746-61.

8. Zhao JL, Huang F, He F, Gao CC, Liang SQ, Ma PF, et al. Forced Activation of Notch in Macrophages Represses Tumor Growth by Upregulating miR-125a and Disabling Tumor-Associated Macrophages. Cancer research. 2016;76(6):1403-15.

9. Chai ZT, Zhu XD, Ao JY, Wang WQ, Gao DM, Kong J, et al. microRNA-26a suppresses recruitment of macrophages by down-regulating macrophage colony-stimulating factor expression through the PI3K/Akt pathway in hepatocellular carcinoma. Journal of hematology & oncology. 2015;8:56.

10. Ma S, Liu M, Xu Z, Li Y, Guo H, Ge Y, et al. A double feedback loop mediated by microRNA-23a/27a/24-2 regulates M1 versus M2 macrophage polarization and thus regulates cancer progression. Oncotarget. 2016;7(12):13502-19.

11. Rahl PB, Lin CY, Seila AC, Flynn RA, McCuine S, Burge CB, et al. c-Myc regulates transcriptional pause release. Cell. 2010;141(3):432-45.

12. Graff JW, Dickson AM, Clay G, McCaffrey AP, Wilson ME. Identifying functional microRNAs in macrophages with polarized phenotypes. The Journal of biological chemistry. 2012;287(26):21816-25.

13. Alzahrani AM, Hanieh H, Ibrahim HM, Mohafez O, Shehata T, Bani Ismail M, et al. Enhancing miR-132 expression by aryl hydrocarbon receptor attenuates tumorigenesis associated with chronic colitis. International immunopharmacology. 2017;52:342-51.

14. Ying X, Wu Q, Wu X, Zhu Q, Wang X, Jiang L, et al. Epithelial ovarian cancer-secreted exosomal miR-222-3p induces polarization of tumor-associated macrophages. Oncotarget. 2016;7(28):43076-87.

15. Li Y, Zhao L, Shi B, Ma S, Xu Z, Ge Y, et al. Functions of miR-146a and miR-222 in Tumor-associated Macrophages in Breast Cancer. Scientific reports. 2015;5:18648.

16. Ren W, Hou J, Yang C, Wang H, Wu S, Wu Y, et al. Extracellular vesicles secreted by hypoxia pre-challenged mesenchymal stem cells promote non-small cell lung cancer cell growth and mobility as well as macrophage M2 polarization via miR-21-5p delivery. Journal of experimental & clinical cancer research : CR. 2019;38(1):62.

17. Hsu YL, Hung JY, Chang WA, Jian SF, Lin YS, Pan YC, et al. Hypoxic Lung-Cancer-Derived Extracellular Vesicle MicroRNA-103a Increases the Oncogenic Effects of Macrophages by Targeting PTEN. Molecular therapy : the journal of the American Society of Gene Therapy. 2018;26(2):568-81.

18. Dang W, Qin Z, Fan S, Wen Q, Lu Y, Wang J, et al. miR-1207-5p suppresses lung cancer growth and metastasis by targeting CSF1. Oncotarget. 2016;7(22):32421-32.

19. Huang F, Chen Z, Chen H, Lu W, Xie S, Meng QH, et al. Cypermethrin Promotes Lung Cancer Metastasis via Modulation of Macrophage Polarization by Targeting MicroRNA-155/Bcl6. Toxicological sciences : an official journal of the Society of Toxicology. 2018;163(2):454-65.

20. Gulei D, Raduly L, Broseghini E, Ferracin M, Berindan-Neagoe I. The extensive role of miR-155 in malignant and non-malignant diseases. Molecular aspects of medicine. 2019;70:33-56.
